# Nutrients Limiting Soybean (*glycine max l*) Growth in Acrisols and Ferralsols of Western Kenya

**DOI:** 10.1371/journal.pone.0145202

**Published:** 2015-12-30

**Authors:** Ludy Keino, Frederick Baijukya, Wilson Ng’etich, Abigael N. Otinga, John R. Okalebo, Ruth Njoroge, John Mukalama

**Affiliations:** 1 Department of Soil Science, University of Eldoret, Eldoret, Kenya; 2 International Institute of Tropical Agriculture (IITA), Dar es Salaam, Tanzania; 3 International Centre for Tropical Agriculture, Nairobi, Kenya; College of Agricultural Sciences, UNITED STATES

## Abstract

Low soybean yields in western Kenya have been attributed to low soil fertility despite much work done on nitrogen (N) and phosphorus (P) nutrition leading to suspicion of other nutrient limitations. To investigate this, a nutrient omission trial was set up in the greenhouse at the University of Eldoret-Kenya to diagnose the nutrients limiting soybean production in Acrisols from Masaba central and Butere sub-Counties, and Ferralsols from Kakamega (Shikhulu and Khwisero sub-locations) and Butula sub-Counties and to assess the effect of liming on soil pH and soybean growth. The experiment was laid out in a completely randomized design with ten treatments viz; positive control (complete), negative control (distilled water), complete with lime, complete with N, minus macronutrients P, potassium (K), calcium (Ca), magnesium (Mg) and sulphur (S) and with, micro-nutrients boron (B), molybdenum (Mo), manganese (Mn), copper (Cu) and zinc (Zn) omitted. Visual deficiency symptoms observed included interveinal leaf yellowing in Mg omission and N addition and dark green leaves in P omission. Nutrients omission resulted in their significantly low concentration in plant tissues than the complete treatment. Significantly (P≤ 0.05) lower shoot dry weights (SDWs) than the complete treatment were obtained in different treatments; omission of K and Mg in Masaba and Shikhulu, Mg in Khwisero, K in Butere and, P, Mg and K in Butula. Nitrogen significantly improved SDWs in soils from Kakamega and Butula. Liming significantly raised soil pH by 9, 13 and 11% from 4.65, 4.91 and 4.99 in soils from Masaba, Butere and Butula respectively and soybean SDWs in soils from Butere. The results show that, poor soybean growth was due to K, Mg and P limitation and low pH in some soils. The results also signify necessity of application of small quantities of N for initial soybean use.

## Introduction

Soybean stands out as the most popular grain legume in the world. Its popularity is attributed to a number of factors related to its composition and productivity. Soybean is a source of the most consumed edible oil and protein source for livestock feeds[1]. Many other soybean products are directly used for human consumption including soymilk, soya sauce, protein extracts and concentrates. Apart from nutritional qualities, soybean yield is higher than other common grain legumes, has relatively few field and storage pests and diseases and has a high nitrogen fixing ability[2]. Cultivation of soybean is gaining interest in Africa following high demand from the booming livestock feed industry (which consumes about 70–80% of soybean produced per year) and need to improve N uptake by the crops which is obtained frombiological nitrogen fixation[3,4,5]. Kenya is not an exception with substantial demand of approximately 150,000 mt yr^-1^[6]. Despite this huge demand, soybean production in Kenya is estimated at 8,000 mtyr^-1^, with 80% of the volume produced in western Kenya. Yield gap in western Kenya remains wide with average yields of 600 kg ha^-1^ against the potential yield of 3,000 kg ha^-1^[5,7].

Low soybean yields in western Kenya are largely attributed to declining soil fertility[7,8]. Adaptive research campaigns initiated across western Kenya by the N2Africa project team to assess the responses of soybean to the application of P and K fertilizers and their combination with inoculants recorded yield increase on only 60% of the sites[9]. An increase in soybean grain yields upon the application of commercial rhizobial inoculants (legume fix) with P fertilizers has also been reported [8]. The yields obtained were however lower (620 kgha^-1^) than the potential yield (yields obtained when the farmers use well adapted, high yielding varieties and better management practices)[5]. In these studies low yields mainly coincided with poor nodulation and a lack of response to P and K fertilizers and inoculants and may indicate that other nutrients may be limiting [9]. The soils not responding to P and K application were grouped as non-responsive. These non-responsive soils include the highly weathered-nutrient-depleted soils (Acrisols and Ferralsols); the majority of which are on land owned by poor farmers.

The low crop yields experienced in these Acrisols and Ferralsols could be attributed to chemical, physical and biological factors. The biological factors involve plant’s genetic variability for nodulation and nitrogen fixation [10]also on the presence of the compatible rhizobial inoculants [11]. Soil physical properties which are of importance in crop production are soil bulk density, water holding capacity, aeration, soil texture and structure[12]. The chemical factors include nutrient limitations which can be caused by intensive cultivation with minimal fertilizer use, nutrient removal via crop harvests, soil erosion, and leaching during heavy rainfall and soil acidity[13]. The minimal fertilizer use in this region can be attributed to lack of money by the farmers to purchase fertilizer and their low expertise on how to use the fertilizers effectively among others. Nutrient deficiencies such as P, Ca, Mg, Mo and K can also be associated with soil acidity. A decrease in soil pH increases the concentration of Fe and Al ions among other cations such as Mn^4+^, H^+^ and Cu^2+^in soil solution[13,14]. These ions cause P sorption through their reaction with phosphate ions to form insoluble compounds[14]. When acidification processes occur in the soil, the hydrogen ion concentration is increased. The hydrogen ion displaces the cations which becomes susceptible to leaching especially during heavy rainfall. [15,16]. This can be the case in western Kenya since most of the soils (about 0.9 million hectares of land) have pH values less than 5.50[17]. Liming, which has been documented as a conventional way of amending acidic soils can benefit these soybean farmers[18]. Given the over aching need to close the yield gap in smallholder farms in western Kenya, there is need to shift the focus to nutrients other than N, P and K which have received the most attention without much increase in yield[19]. This has been linked to the results from the sites where previous experiments had been carried out by an N2Africa research team through International Centre of Tropical Agriculture (CIAT), to test the effects of P and K fertilizers applied together with the inoculants on soybean yields. These resulted to lower grain yields of soybean compared to the potential yields. This study therefore aimed at investigating the factors causing this non-responsiveness by establishing other nutrients limiting soybean production and also to assess the effects of liming on soil pH and soybean performance.

## Materials and Methods

### Study soils, sampling and analysis

The soils that were used in the experiment were collected from five distinct locations representing major soybean growing areas in western Kenya (Table 1). The fields in which these soils were obtained were privately owned. The permission to collect the soils from these lands was granted by the farmers who owned them. These sites were identified with the help of a research scientist from International Centre for Tropical Agriculture(CIAT) Maseno -Kenya. Field experiments had been carried out in these sites by an N2Africa research team through CIAT to test the effects of P and K fertilizers with inoculants on soybean where they showed very little response. The soil types in these sites were sourced from the farm management handbook of western Kenya [20]. The soil types were classified according to the international FAO soil classification system. The soils from Masaba central were classified as chromic Acrisols. These soils are well drained, deep to very deep, red to dark brown, friable, sandy to clay. The soil type from Kakamega (Khwisero and Shikhulu sub-location) is Ferralo-humic Acrisols. These soils are well drained, deep to very deep, yellowish red to dark reddish brown, friable to firm, sandy clay, with an acid or thick acid humic top soil. The soils from Butula were classified as Rhodic Ferralsols and their characteristics are; well drained, moderately deep to deep, dark reddish brown to strong brown, friable sandy, clay loam to clay. From each site approximately 60 kg of soil were collected by taking the top soil (0–20 cm) at 20 points in a zig zag manner using a hand hoe. The 20 soil portions from one site were mixed to come up with a composite representative sample. The soils were then air-dried and sieved to pass a 5 mm. From the remaining soils of each location, a sub-sample of about 250 g was taken for chemical and physical characterization at the Crop Nutrition Laboratory in Nairobi-Kenya for pH, total N, extractable P, organic carbon, exchangeable cations (Ca, K, Mg) and particle size following methods described in [21]. These characteristics are shown in Table 2. Soils from all the sites were strongly acidic (4.50 to 5.00) except for the soils from Kakamega (Khwisero sub-location) which were moderately acidic (5.08)[17]. Percentage nitrogen levels were moderate for all the soils (0.12 to 0.25)[21]. The levels of extractable P were below the critical level (< 10 mg/kg) in all the soils while the exchangeable cations were of low to moderate levels[21].

**Table 1:** Location and characteristics of the sites where experimental soils were sampled from.

Site	Sub-location	District	Latitude	Longitude	Altitude (m asl)	Soil type	Average Rainfall and temperature (per annum)
Masaba Central	Masaba	Butere	034^0^ 27’ 38.2”E	00^0^ 11’59.9”N	1331	Chromic Acrisols	1685–1882 mm, 13.9–30.2°C
Kakamega	Khwisero	Kakamega south	034^0^ 40’ 20.4”E	00^0^ 12’ 26.0” N	1488	Ferralo-humic Acrisols	1730–1929 mm, 14.1–27.1°C
Kakamega	Shikhulu	Kakamega South	034^0^ 40’ 05.4” E	00^0^ 12’ 14.6” N	1508	Ferralo-humic Acrisols	1730–1929 mm, 14.1–27.1°C
Butere	Emutsatsa	Butere	034^0^ 27’ 56.9” E	00^0^ 11’ 51.35” N	1344	Chromic Acrisols	1685–1882 mm, 13.9–30.2°C
Butula	Bukhalalire	Butula	034^0^ 16’ 48.9” E	00^0^ 19’ 11.8” N	1219	Rhodic Ferrasols	1790–2016 mm, 15.8–28.6°C

All the sites were under the humid lower midland zones Source:[20]

**Table 2:** Physico- chemical properties of the soils used in the experiment.

**Parameters**	**Masaba**	**Kakamega (Khwisero)**	**Kakamega (Shikhulu)**	**Butere**	**Butula**
**pH (water)**	4.65	5.08	4.99	4.91	4.99
**Total N (%)^a^**	0.13	0.24	0.20	0.13	0.15
**Available P (mg/kg)^b^**	4.43	7.27	4.58	2.11	6.18
**Organic C^a^**	1.50	3.13	2.59	1.25	1.58
**C:N ratio**	11.53	13.04	12.95	9.62	10.53
**CEC^c^**					
**K (cmol_c_/kg)**	0.16	0.18	0.17	0.10	0.16
**Ca (cmol_c_/kg)**	1.10	4.34	3.22	1.03	2.49
**Mg (cmol_c_/kg)**	0.51	1.70	0.93	0.39	0.98
**TEXTURE^d^**					
**Sand (%)**	54	48	52	60	58
**Clay (%)**	30	32	32	18	24
**Silt (%)**	16	20	16	22	14
**Textural class**	Sandy clay loam	Sandy clay loam	Sandy clay loam	Sandy loam	Sandy clay loam

^a^Vario max CN[47]

^b^Fox and Kamprath[48]

^c^ ICP-OES (Perkin Elmer, Inc.)

^d^ Based on Stokes law

### Nutrient treatments

The composition of the nutrient solutions were based on Hoagland half strength solution[22], according to specific requirements of soybean. The ion concentrations were in milli-moles per litre (mmol/l) for the macro-nutrients and micro-moles per litre (μmol/l) for the micro-nutrients. The macro-nutrients tested were P, K, Mg, Ca, S, and micronutrients (B, Mo, Mn, Cu and Zn) (Table 3). The treatments included one positive control (complete nutrient solution), negative control (only distilled water), 5 treatments where one element was omitted from the nutrient solution, one treatment where the micro-nutrient mixture was omitted from the nutrient solution, one treatment with an additional nitrogen source in the nutrient solution and one treatment with lime addition to the soils (Table 4). The purpose of the complete plus N treatment was to assess whether the poor performances of plants with omitted elements was due to the element in question alone or to nitrogen limitation. In literature e.g.[11,23], it was documented that poor performance of soybean plants in nutrient omission trials was also due to nitrogen deficiencies. The inclusion of the lime treatment stemmed from the fact that all the experimental soils were acidic. Lime and plus nitrogen treatments were added only to the complete treatment. The lime requirements of the soils were determined using the procedure described by[24], with the target pH of 6.2. This method relies on the initial soil pH and soil texture of the test soil. This is followed by referring to the appropriate tables based on the target pH and crop to be grown. From these tables, the calcium carbonate equivalency and the actual amount to be applied are obtained[24]. The liming material that was used is Koru lime with the following composition; CaO–burnt lime (20.8%), MgO (1.06%), Fe_2_O_3_ (0.29%), Al_2_O_3_ (1.2%) and SiO_2_ (0.42%).

**Table 3:** Nutrient salts and rates used to prepare nutrient solutions.

**Element**	**Salt**	**Rate of application**
**Macro-nutrients**		**(millimols/l)**
**N and Ca**	Ca(NO_3_)_2_.4H_2_O/NH_4_NO_3_/ CaCl_2_.2H_2_O	N = 7.5
		Ca = 2.5
**P**	H_3_PO_4_	P = 0.5
**Mg, S and K**	MgSO_4_/K_2_SO_4_	Mg = 1
		SO_4_^-^ = 1
		K = 3
**Micro-nutrients**		**(Micromoles/l)**
**B**	H_3_BO_3_	7.13
**Mo**	Na_2_MoO_4_-2H_2_O	0.01
**Zn**	ZnSO_4_	0.96
**Cu**	CuSO_4_	1.04
**Mn**	MnCl_2_	7.4

Source:[49]; modified from Hoagland solution[22].

**Table 4:** Treatments used in the experiment.

No.	Treatments	Macro-nutrients						Micro-nutrients	Lime
		N	P	K	Mg	Ca	S		
**1**	Complete	-	+	+	+	+	+	+	-
**2**	Complete plus lime	-	+	+	+	+	+	+	+
**3**	Control	-	-	-	-	-	-	-	-
**4**	P omitted	-	-	+	+	+	+	+	-
**5**	Complete plus N	+	+	+	+	+	+	+	-
**6**	K omitted	-	+	-	+	+	+	+	-
**7**	Mg omitted	-	+	+	-	+	+	+	-
**8**	Ca omitted	-	+	+	+	-	+	+	-
**9**	S omitted	-	+	+	+	+	-	+	-
**10**	Micro-nutrients omitted	-	+	+	+	+	+	-	-

**KEY:** + (Nutrient included in the nutrient solution)—(Nutrient omitted from nutrient solution)

### Set up of the experiment

**Fig 1:**
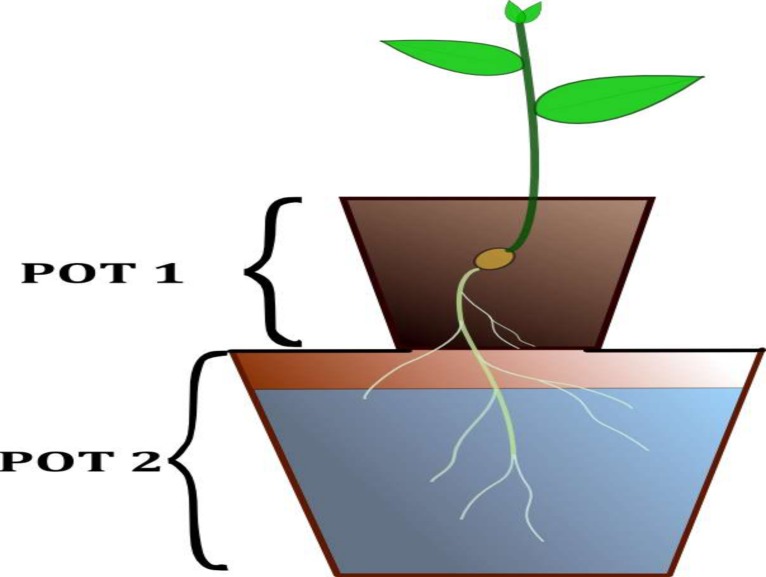
Illustration of the set up of the double pot experiment.

Assessment of limiting nutrients was carried out in the greenhouse at the University of Eldoret located at 0° 34' N and 35° 18' E. The experiment adopted the so called double pot technique[25]. This method offers an easy and rapid means of identifying the nutrients that are in short supply in the soils. In this technique, two pots are used whereby the upper pot (pot 1, Fig 1) has a gauze fitted at the bottom and is filled with soil. The lower pot (pot 2, Fig 1) is filled with nutrient solution and has a lid to support the upper pot. A space of approximately 1 cm is left between the bottom of the upper pot and the nutrient solution to allow oxygen supply to the plant roots. The plant is therefore provided with two sources of nutrients (test soil and nutrient solution) for its simultaneous uptake. It is during the initial stage of growth (germination) that the plant obtains nutrients from the soil alone. Seeds or seedlings are planted in the soil and as the plants grow the roots pass through the gauze and reach the nutrient solution in the lower pot. When a nutrient is omitted from the nutrient solution, the plant can take it up from the soil only. The absence of an element can be seen from the deficiency symptoms developed such as limited growth and leaf chlorosis. The symptoms can be visible in the early growth stages and thus can be used to draw conclusions for fertilizer recommendations. The technique ensures those nutrients applied such as P and K are not fixed by the soil as it would be the case if soil alone (single pot) is used. In this experiment, the upper pots were provided by parts of a common sewage pipe with 9 cm diameter. A mesh was cut into small pieces and tied to their bottom, to prevent the soil from falling into the solution, but providing passage for the roots. Small plastic pots of 2 l volume served as the bottom pots.

### Experimental design

The experiment was laid out in a completely randomized design (CRD) whereby the treatments were replicated four times. The set up was repeated three times to allow for the destructive sampling at three time intervals. There were 120 pots per soil (10 treatments x 4 replications x 3 pots per replication) giving a total of 600 pots for the five soils. The treatments were randomly allocated to the pots (experimental units). The experimental factors were the soils and the nutrient treatments. The pots were rotated two times a week to be able to eliminate any biasness from one side of the greenhouse

### Management of experiment

Soybean cultivar TGX 1740-2F (SB19) was planted in the experiment (3^rd^ May to 2^nd^ June 2013) with the temperatures in the greenhouse ranging from a minimum of 18.3 to a maximum of 35.8°C. This cultivar was chosen because it is commonly grown in western Kenya due to its early maturity and promiscuity in nitrogen fixation. From previous experiments this cultivar has also had low yields after application of inoculants and P and K fertilizers. The seeds were obtained from CIAT Maseno-Kenya. They were inoculated using the commercial Biofix inoculant containing *Bradyrhizobium japonicum* strain USDA 110 which was acquired from MEA Fertilizers Ltd-Kenya. Three seeds were sown per pot and were thinned to single uniform plants per pot 7 days after emergence. The thinned plants were left in the specific pots and the soils in the pots were covered with gravel to reduce evaporation. Prior to establishing the experiment, the field capacity of each of the soils was established by saturating the soils with water and covering them using perforated polythene papers. They were then weighed and then left for 48 hours in the greenhouse. The difference between the weight of the container plus saturated soil and that of the container plus soil after 48 hours represented the field capacity. During the experiment the pots were watered daily with distilled water and the soils maintained at field capacity. The nutrient solution was renewed at 3 weeks after planting by pouring out the old solution and adding a new solution into the container. One molar HCl was used to regulate the pH of the alkaline nutrient solutions while 0.1 molar NaOH was used to regulate the pH of the acidic nutrient solution (-K). The target pH was 6.3 to 6.5.

### Data collection

Data were collected on stem length and shoot dry weight.These measurements were taken 14, 21 and 28 days after emergence (DAE). The destructive sampling was done at the three time intervals to help in determination of the relative growth rate and nutrient sufficiency quotients. The plants were oven dried at 65°C for 48 hrs to determine shoot dry weight. After drying, the plant samples at the final harvest were ground. The four samples from each replicate representing one treatment were aggregated. They were then sent to the Crop Nutrition Laboratory Services Ltd, Nairobi-Kenya for elemental analysis using inductively coupled plasma optical emission spectrometry (ICP-OES, Perkin Elmer Inc). The extraction solution used was Mehlich 3 Extractant: (0.2 N CH_3_COOH + 0.25 N NH_4_NO_3_ + 0.015 N NH_4_F + 0.013 N HNO_3_ + 0.001 M EDTA).The analysis carried out established, N, P, K, Ca, Mg, Mn, B, Zn and Cu concentrations in the plant tissue samples within the soil types. Analysis for S and Mo was not carried out because during the time of analysis, machines for analysis were broken down. Visual observations for any deficiency symptoms were recorded daily from 8 days after emergence to the end of the experimental period.

### Determination of relative growth rate and nutrient sufficiency quotients (SQ’s)

The relative growth rate based on shoot dry weight was calculated between two time intervals 14–21 DAE and 21–28 DAE as shown in Eq 1. This reflected the net growth of the plants growing on different nutrient solutions. The relative growth rates obtained were then used to calculate the nutrient sufficiency quotients, to show the relationship between nutrient treatments and the complete treatment. This was done as shown in Eq 2 i.e. dividing the relative growth rate of the element in question to that of the complete treatment. The SQ’s were then multiplied by 100 to reflect the percentage growth of a given treatment compared to the complete treatment. The nutrient treatments with SQs less than 50% were considered to be more limiting, those more than 50% were considered to be less limiting[11,23].The formulas used in calculation of relative growth rate and finally the nutrient sufficiency quotients were; [25].

$$Rs=\frac{lnS2-lnS1}{t2-t1}Rs$$ 1

Where; Rs = Relative growth rate, S = shoot dry weights in g, t = time in days and ln = natural logarithm

The following formula was used to calculate SQ’s;[23,26].

$$SQx=\frac{{Rs}_{x}}{{Rs}_{C}}\text{SQ}$$ 2

Where; SQ_x_ = Sufficiency quotient for x, where x is the nutrient element in question, (RS)-x = Relative growth rate of plants growing in nutrient solutions with x (nutrient element in question) omission and RS(C) = Relative growth rate of plants growing on complete nutrient solution.

### Statistical analysis

Analysis of variance was done to compare the effects of the different treatments on growth and performance of soybean using Genstat 12^th^ edition. The effects of the different treatments were compared by computing the least square means and their standard errors of the difference (SED). All the single nutrient treatments were compared to the complete treatments[25] using the least significant differences to establish their extent of limitation. If a treatment was significantly lower than the complete treatment, then it was considered to be limiting in the soils. Significance of the difference was evaluated at P≤ 0.05 significant level. Each soil was analyzed separately to be able to identify limiting nutrients specific to each soil. Data analysis for shoot nutrient concentration was done irrespective of the soil types (across the soils) whereby the soils served as replicates.

## Results

### Visual observations

**Fig 2:**
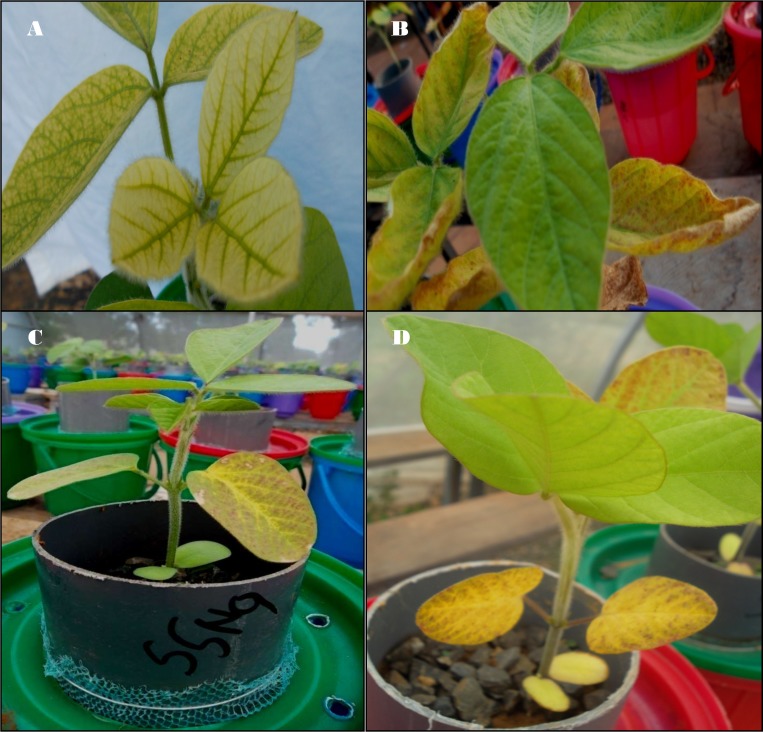
Nutrient deficiency symptoms noted during plant growth. (A) Mg deficiency in plus nitrogen treatment in Ferralsols from Kakamega (Shikhulu Sub-location), (B) K deficiency in minus K treatments in Ferralsols from Kakamega (Shikhulu sub-location), (C) P deficiency in minus magnesium treatments in Ferralsols from Butula, (D) Micro-nutrient deficiencies in minus micro-nutrient treatments in Acrisols from Masaba Central.

**Fig 3:**
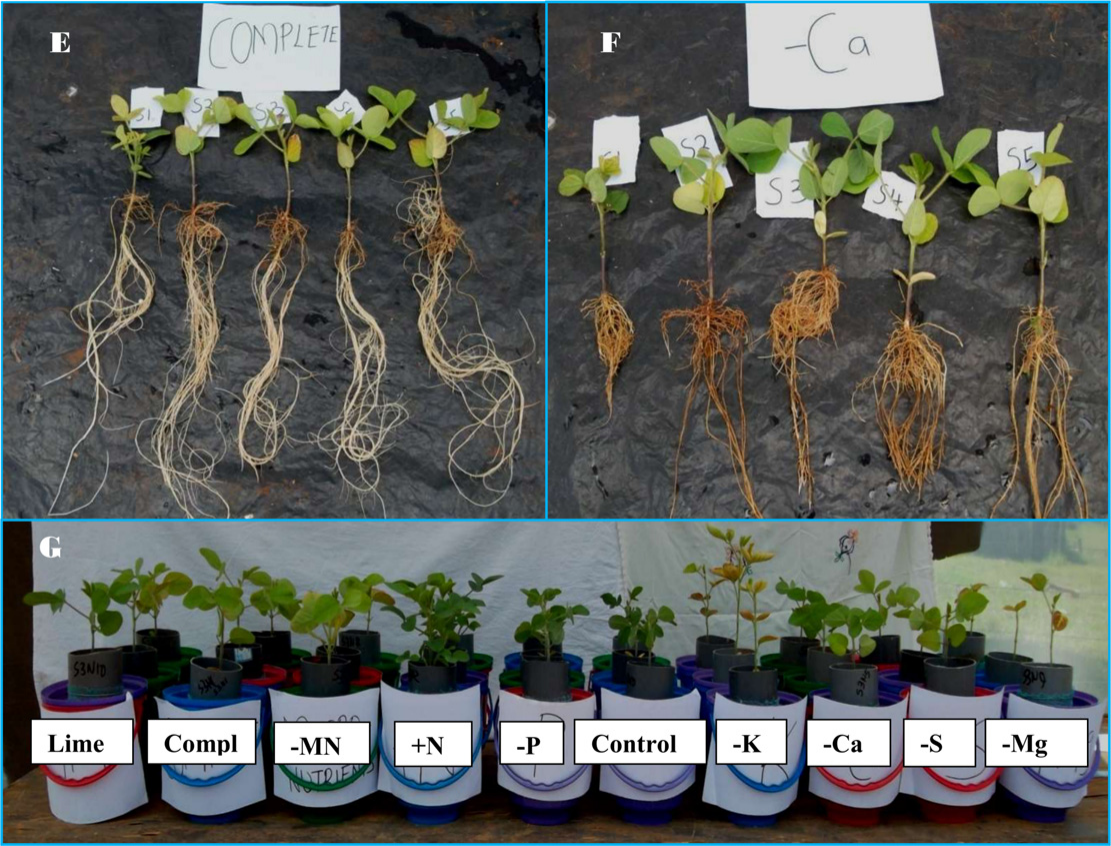
Soybean plants at the time of harvest. (E) Plants growing on complete nutrient solution on different soils, (F) Plants growing on minus calcium nutrient solution on different soils, (G) Plants growing on different nutrient treatments in Ferralsols from Kakamega (Shikhulu sub-location). Compl–complete treatment, MN- minus micro-nutrients.

The most common deficiency symptom observed was that of Mg where there was interveinal leaf yellowing especially in treatments of plus nitrogen and a few of Mg omitted treatments across the soils (Fig 2A). Deficiencies of K were also noted mainly on older leaves of K omitted treatments and also on several other treatments. These were characterized by leaf yellowing with tissue necrosis along the leaf margins (Fig 2B). Early leaf drop was observed in plants growing in the treatments with both Mg and K deficient treatments, and this led to low shoot dry weights being recorded by the same treatments, especially at the final harvest. Plants growing in the control (only distilled water) and in the treatments where P and Ca were omitted maintained green leaf colour to the end of the experimental period. Other treatments (complete, minus sulphur, minus micro-nutrients and complete plus lime) had pale yellow leaves at the time of harvesting (Fig 3G) indicating possible deficiencies of nitrogen.

**Fig 4:**
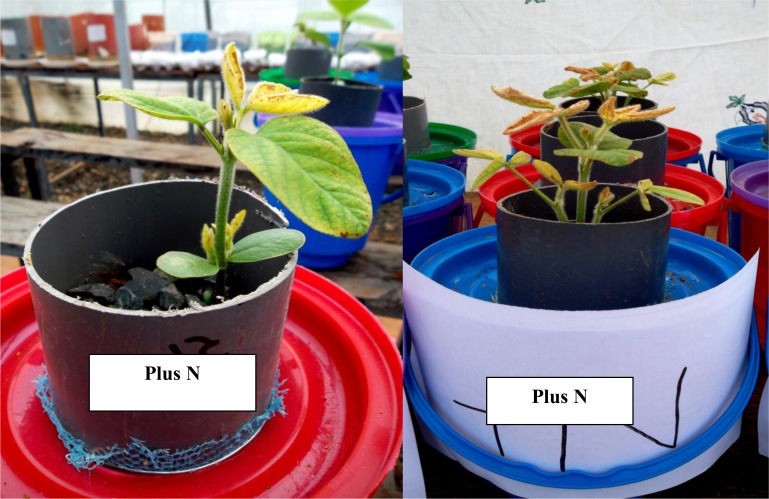
Scorching and necrosis of leaves in nitrogen added treatments in Acrisols from Masaba central.

There were well-developed rooting systems in all the treatments (Fig 3E) except in calcium omitted treatments and also in some of the plus nitrogen treatments (Fig 3F). Addition of nitrogen to the nutrient solution had a variable response across the soils. Ferralsols from Kakamega and Butula produced very leafy green plants (Fig 3G) while in the Acrisols from Masaba central and Butere, the foliage was less and there was a scorching characteristic at the tips and edges of the leaves (Fig 4).

### Trends of shoot nutrient concentration as influenced by the treatments in different soils

Table 5 shows the shoot nutrient concentration in the soybean plants across the soils. Omission of P, Mg, K, and Ca resulted to their significantly (P ≤ 0.05) low concentration than the complete treatment. Omission of micro-nutrients from the nutrient solution resulted into significantly lower B and Zn in the plant tissues than the complete treatment across the soils. Application of N to the nutrient solution led to its significantly higher concentration in the plant tissues than the complete treatment across the soils. It also led to significantly low concentration of P and Ca than the complete treatment. Lime application to the soils did not differ significantly (P ≤ 0.05) from the complete treatment in all the treatments analyzed. The control treatment had significantly lower P, K, Ca and B concentration in their plant tissues than the complete treatment.

**Table 5:** Effects of the different treatments on shoot nutrient concentrations across the different soils.

Treatments	%P	%N	%Mg	%K	%Ca	B (ppm)	Cu (ppm)	Mn(ppm)	Zn (ppm)
Control	0.15^a^	2.51^ab^	0.34^b^	0.83^a^	0.68^b^	24.90^a^	3.32^ab^	428^a^	49.40^cd^
Minus Ca	0.30^ab^	2.30^ab^	0.38^b^	1.99^b^	0.39^a^	26.52^a^	1.88^a^	410^a^	31.56^a^
Minus K	1.42^e^	2.96^b^	0.59^c^	1.18^a^	1.68^e^	42.56^e^	5.52^d^	545^a^	64.5^e^
Minus Mg	1.40^e^	2.95^b^	0.24^a^	2.99^d^	1.56^de^	50.30^f^	4.95^bcd^	459^a^	59.84^de^
Minus micro-nutrients	0.83^d^	2.04^ab^	0.39^b^	2.49^bcd^	1.40^d^	28.84^ab^	3.16^ab^	409^a^	32.68^a^
Minus P	0.21^a^	2.78^ab^	0.36^b^	2.01^b^	0.93^c^	32.46^bcd^	3.14^ab^	476^a^	43.58^abc^
Minus S	0.66^cd^	1.98^a^	0.38^b^	2.26^bc^	1.40^d^	33.94^bcd^	3.44^abc^	430^a^	34.98^ab^
Plus nitrogen	0.52^bc^	4.06^c^	0.35^b^	2.60^cd^	1.04^c^	30.54^abc^	5.25^cd^	436^a^	40.62^abc^
Complete	0.79^d^	2.15^ab^	0.40^b^	2.46^bc^	1.45^d^	34.92^cd^	4.33^bcd^	387^a^	46.86^bc^
Complete plus lime	0.74^cd^	2.22^ab^	0.36^b^	2.27^bc^	1.58^de^	37.32^de^	3.58^abc^	156^a^	35.70^ab^
**Grand mean**	0.703	2.6	0.3772	2.107	1.211	34.23	3.86	414	44
**S.E.D**	0.1293	0.468	0.0378	0.252	0.0934	2.836	0.951	172.9	6.24
***F*.*Probabilit*y**	< .001	0.002	< .001	< .001	< .001	< .001	0.011	NS	< .001
**C.V.**	29.1	28.5	15.8	18.9	12.2	13.1	39	66.1	22.5

Similar letters in each column shows non-significant difference to fisher’s protected LSD (P ≤ 0.05). conc.–concentration.

Elimination of Ca from the nutrient solution led to significantly lower accumulation of P, Ca, B, Cu and Zn in the shoots compared to the complete treatment across all the soils. Omission of Mgand Kfrom the nutrient solution led to significantly (P≤0.05) higher concentration of P, K, B Zn and P, Mg, Ca, B and Zn respectively compared to the complete treatment across all the soils (Table 5).

### Effects of treatments on root dry weights at final harvest

The treatments differed significantly (P ≤ 0.001) from each other in terms of root dry weight in soils from all the sites (Table 6). The control treatment had significantly lower root dry weights than the complete treatment in soils from all the sites. In soils from Masaba central, omission of Ca, K, Mg and addition of N in the nutrient solution resulted in significantly lower root dry weights than the complete treatment. Omission of Mg, K, Ca, and addition of N to the nutrient solution in Kakamega (Khwisero Sub-location) had significantly lower root dry weights than the complete treatment. In Kakamega (Shikhulu Sub-location) omission of Ca, K and Mg significantly reduced the root dry weights than the complete treatment while in Butere, this occurred with the omission of K and addition of N to the nutrient solution. Omission of K, Mg, addition of N to the nutrient solution and addition of lime to the soils significantly reduced the root dry weights than the complete treatment in soils from Butula. Application of lime in Acrisols from Masaba and Butere significantly increased root dry weights than the complete treatment (Table 6).

**Table 6:** Root dry weights (g/plant) for the different treatments in soils from different sites.

Treatments	Masaba	Kakamega 1	Kakamega 2	Butere	Butula	Across soils
Control	0.11^a^	0.16^b^	0.14^bc^	0.09^b^	0.14^bc^	0.13^bc^
Minus Ca	0.11^a^	0.15^ab^	0.15^bc^	0.14^cd^	0.18^cd^	0.15^c^
Minus K	0.08^a^	0.15^ab^	0.08^a^	0.04^a^	0.06^a^	0.08^a^
Minus Mg	0.10^a^	0.11^a^	0.10^ab^	0.14^cd^	0.09^ab^	0.11^b^
Minus MN	0.18^b^	0.23^d^	0.18^cd^	0.21^e^	0.17^cd^	0.19^de^
Minus P	0.18^b^	0.21^cd^	0.24^e^	0.12^bcd^	0.18^cd^	0.18^d^
Minus S	0.20^b^	0.22^cd^	0.19^cde^	0.21^e^	0.18^cd^	0.20^de^
Plus N	0.08^a^	0.18^bc^	0.23^e^	0.11^bc^	0.16^c^	0.15^c^
Complete	0.19^b^	0.24^d^	0.23^de^	0.16^d^	0.23^d^	0.21^e^
Complete+Lime	0.25^c^	0.21^cd^	0.19^cde^	0.26^f^	0.16^c^	0.21^e^
*F*. *Probability*	< .001	< .001	< .001	< .001	< .001	< .001
S.E.D	0.0286	0.02201	0.02769	0.217	0.0295	0.0128
% CV	27.2	16.9	22.8	21.1	26.9	25.2

Similar letters in each column shows non-significant difference to fisher’s protected LSD (P ≤ 0.05). Means are compared between the different treatments within the same soil (along the column). MN–micronutrients. Kakamega1 (Khwisero sub-location), Kakamega2 (Shikhulu sub-location)

### Effects of treatments on shoot biomass accumulation at final harvest

The treatments differed significantly (P ≤ 0.001) in the soils from all the sites with respect to the shoot dry weights. The control treatments had significantly lower shoot dry weights compared to the complete treatment in all the soils except those from Kakamega (Khwisero Sub-location) (Table 7). In Acrisols from Masaba central, omission of K, Mg, and, Ca resulted in significantly lower shoot dry weights compared to the complete nutrient treatment. Shoot dry weight of the S, P, micro-nutrients omitted treatments, plus lime and nitrogen added treatments were not significantly different from the shoot dry weight of the complete nutrient treatment. When Mg was excluded from the nutrient solution in Ferralsols from Kakamega (Khwisero sub-location), there was significantly lower shoot dry weights compared to that of the complete treatment. There was no significant difference in shoot dry weights between the complete treatment and when K, Ca, P, S and micro-nutrients were omitted from the nutrient solutions. Addition of Nitrogen to the nutrient solution increased the shoot dry weights significantly than the complete treatment. Lime addition to the soils on the other hand did not differ significantly than the complete treatment (Table 7).

**Table 7:** Shoot dry weights (g/plant) for the different treatments in soils from the different sites.

Treatments	Masaba	Kakamega 1	Kakamega 2	Butere	Butula	Across soils
Control	0.28^b^	0.38^ab^	0.41^ab^	0.22^a^	0.38^bc^	0.33^bc^
Minus Ca	0.37^bc^	0.68^cd^	0.57^cd^	0.56^de^	0.64^f^	0.56^ef^
Minus K	0.16^a^	0.49^abc^	0.28^a^	0.15^a^	0.20^a^	0.25^a^
Minus Mg	0.29^b^	0.33^a^	0.29^a^	0.25^ab^	0.29^ab^	0.29^ab^
Minus MN	0.41^cd^	0.70^d^	0.48^bc^	0.49^cd^	0.43^bcd^	0.50^de^
Minus P	0.41^cd^	0.40^ab^	0.45^bc^	0.28^ab^	0.38^bc^	0.38^c^
Minus S	0.43^cd^	0.62^cd^	0.48^bc^	0.48^cd^	0.48^cde^	0.50^d^
Plus N	0.42^cd^	1.01^e^	1.06^e^	0.37^bc^	0.86^g^	0.74^g^
Complete	0.52^de^	0.57^bcd^	0.59^cd^	0.37^bc^	0.57^def^	0.52^de^
Complete+Lime	0.61^e^	0.54^bcd^	0.64^d^	0.63^e^	0.61^ef^	0.61^f^
*F*. *Probability*	< .001	< .001	< .001	< .001	< .001	< .001
S.E.D	0.0577	0.0981	0.0695	0.0685	0.0702	0.0330
% CV	20.9	24.3	18.7	25.5	20.5	22.2

Similar letters in each column shows non-significant difference to fisher’s protected LSD (P ≤ 0.05). Means are compared between the different treatments within the same soil (along the column). MN–micronutrients. Kakamega1 (Khwisero sub-location), Kakamega2 (Shikhulu sub-location)

In Ferralsols from Kakamega (Shikhulu sub-location), omission of K, and Mg resulted in significantly lower shoot dry weights compared to the complete treatment (Table 7). There were no significant differences from the complete treatment when Ca, P, S and micro-nutrients were omitted from the nutrient solution. Addition of nitrogen to the nutrient solution significantly increased the shoot dry weights compared to the complete treatment while lime addition to the soils did not differ significantly from the complete treatments (Table 7). In Acrisols from Butere, significantly lower shoot dry weights were obtained when K was omitted from the nutrient solution. Omission of Ca resulted in significantly higher shoot dry weights than the complete treatment (Table 7). Omission of S, micro-nutrients and P resulted in shoot dry weights that were not significantly different from the complete treatment. The shoot dry weights of the nitrogen added nutrient solutions did not differ significantly from the complete treatment while lime addition to the soils significantly increased the shoot dry weights than the complete treatment (Table 7). When K, P and Mg were omitted from the nutrient solution in Ferralsols from Butula, there was significantly lower shoot dry weights compared to the complete treatment. Omission of Ca, micro-nutrients and S resulted in shoot dry weights that were not significantly different from that of the complete treatment. The shoot dry weights of the nitrogen added nutrient solutions were significantly higher than those of the complete treatment while those of lime application did not differ significantly (Table 7).

Omission of K resulted in significantly lower shoot dry weights than the control (distilled water only) treatment in Acrisols from Masaba central. It was not significantly different from the control treatment in Acrisols from Butere and Ferralsols from Kakamega (Khwisero and Shikhulu sub-location). Omission of Mg did not differ significantly from the control treatment in terms of shoot dry weights in the soils from all the sites (Table 7).

### Nutrient sufficiency quotients

The sufficiency quotients were multiplied by 100 to show the percentage growth of a nutrient treatment compared to the complete treatment. From this experiment, omission of Ca from the nutrient solution in Ferralsols from Kakamega (Shikhulu sub-location), micro-nutrients in Ferralsols from Kakamega (Khwisero sub-location) and Acrisols from Butere and S omission from the nutrient solution in the Ferralsols from Kakamega (Khwisero sub-location) and Butula had sufficiency quotients greater than 100% (Table 8). Omission of Mg and K from the nutrient solution led to lowest sufficiency quotients in all the Acrisols and Ferralsols. The sufficiency quotients of minus K were negative in Ferralsols from Butula while those of minus Mg were negative in Ferralsols from Kakamega (Shikhulu sub-location) (Table 8). The control treatment had low sufficiency quotients in Acrisols from Masaba central and Butere but these values were high than those of minus Mg and minus K. In other soils, they had high sufficiency quotients, even greater than P omitted treatments in Ferralsols from Kakamega (Khwisero and Shikhulu sub-locations) and in Butula. They were greater than minus Ca and minus micro-nutrients in Ferralsols from Butula (Table 8).

**Table 8:** Percent nutrient sufficiency quotients for different treatments and site combination based on shoot dry weights.

		Kakamega			
SITE/TREATMENT	Masaba	Khwisero	Shikhulu	Butere	Butula
Control	42	85	74	42	96
Minus Ca	85	97	113	79	94
Minus K	18	64	48	1	-4
Minus Mg	35	28	-1	50	30
Minus MN	69	126	77	124	92
Minus P	68	68	71	49	82
Minus S	78	122	91	86	106
Plus N	61	178	145	115	110
Complete	100	100	100	100	100
Complete plus lime	83	82	84	129	112

Kakamega1 (Khwisero sub-location), Kakamega2 (Shikhulu sub-location)

Lime addition to the soils led to higher nutrient sufficiency quotients than the complete treatment in Acrisols from Butere and Ferralsols from Butula. In Acrisols from Masaba central and Ferralsols from Kakamega (Khwisero and Shikhulu Sub-locations) the sufficiency quotients of the limed treatments were lower than those of the complete treatments. Nitrogen application to the nutrient solution increased the nutrient sufficiency quotients compared withthe complete treatment in all the soils except in those from Masaba central (Table 8).

### Effects of the lime application on soil pH

The treatments differed significantly (P ≤ 0.05) in terms of soil pH in all the soils except in Ferralsols from Kakamega (Khwisero and Shikhulu sub-locations). Application of lime to the Acrisols from Masaba central and Butere and Ferralsols from Butula raised the soil pH significantly (P ≤ 0.05) compared to the other treatments except minus Mg in Ferralsols from Butula (Table 9).

**Table 9:** Effects of the different treatments on soil pH of the soils from different sites.

Treatments	Masaba	Kakamega1	Kakamega2	Butere	Butula	Across soils
Control	4.55^a^	5.12^a^	5.16^a^	4.81^a^	5.13^b^	4.95^bc^
Minus Ca	4.56^a^	5.25^a^	5.14^a^	5.12^b^	5.03^b^	5.02^c^
Minus K	4.60^a^	5.12^a^	5.09^a^	4.80^a^	5.05^b^	4.93^bc^
Minus Mg	4.67^a^	5.19^a^	5.06^a^	4.85^a^	5.20^bc^	4.99^c^
Minus MN	4.68^a^	5.05^a^	4.75^a^	4.91^ab^	4.98^b^	4.87^b^
Minus P	4.70^a^	5.13^a^	4.91^a^	4.97^ab^	4.91^ab^	4.92^bc^
Minus S	4.66^a^	5.06^a^	5.13^a^	4.84^a^	4.96^ab^	4.93^bc^
Plus N	4.54^a^	5.02^a^	4.81^a^	4.78^a^	4.63^a^	4.75^a^
Complete	4.64^a^	5.14^a^	5.18^a^	4.89^a^	4.91^ab^	4.95^bc^
Plus lime	5.07^b^	5.30^a^	5.24^a^	5.41^c^	5.53^c^	5.31^d^
**S.E.D**	0.0882	0.1081	0.1728	0.0974	0.1543	0.0576
**F. Probability**	0.005	NS	NS	0.002	0.013	< .001
**C.V.**	1.9	2.1	3.4	2	3.1	2.6

Statistical analysis and treatment comparisons done per soil (along the column). Means followed by the same letter are not significantly different (LSD, P ≤ 0.05) MN–micronutrients. Kakamega1 (Khwisero sub-location), Kakamega2 (Shikhulu sub-location)

## Discussion

### Visual observations and plant tissue nutrient concentrations in different soils

Hydroponic systems have been frequently used to study the effects of mineral nutrient deficiencies on plant growth and physiology. This is because they are important in identification of the visual symptoms for diagnostic purposes[27]. In the current study, several deficiency symptoms were noted in the double pot experiment which was used to mimic the hydroponic systems and thus helped in identifying the nutrients limiting in the test soils.The interveinal yellowing of the leaves can be attributed to magnesium deficiency[28]. This can be related to the low Mg concentration in plant tissues growing in magnesium omitted treatments. These values were below the sufficiency ranges (0.3 to 0.6%) for soybean plant at early growth stages [29]. This indicated that the magnesium levels in the soils were low (as shown by the initial soil analysis). Since the element was not provided by the nutrient solution, there was insufficient amount for plant uptake. This treatment also accumulated significantly higher amounts of other nutrients such as, P, K, B and Zn than most of the treatments (Table 5). The low accumulation of Mg in plant tissues growing in Mg-omitted treatments and high accumulation of other elements may suggest that the poor performance of the plants indicates that this nutrient is limiting in the soils.

The plants grown on K-omitted nutrient solution had their older leaves turning yellow with tissue necrosis along the leaf margins, a factor that could be attributed to K deficiency[30]. The concentration of K in the shoot tissues of the plants growing in K-omitted treatments were lower compared to the other nutrients. These concentrations were below the sufficiency ranges (1.7 to 2.5%) for soybean plant tissues at early growth stages[29]. The same plants also had significantly high concentration of other elements such as P, Mg, Ca, Band Zn (Table 5). This shows that K may be limiting in the soils because of high concentration of other nutrients and thus growth is limited by low K.Low K uptake can also be attributed to low concentration of K in the soils. Similar results were found out when working with non-responsive soils in Zimbabwe using a double pot experiment[11]. The presence of other elements in high concentrations in K omitted treatments can be attributed to K competitive ability especially for Ca and Mg[31]. This competition occurs because there are a limited number of ion carrier sites on the root plasma membrane and thus the ions of the same ion strength can out compete each other for the sites[32].

Apart from the dark green leaves and stunted plants grown in minus P treatments, some plants exhibited interveinal reddening which is associated with P deficiency in soybean plants in some instances[33]. This can be evidenced by lower P concentrations in plant tissues growing in P omitted treatments. These concentrations were below the sufficiency ranges (0.3 to 0.6%) required for soybean at early growth stages[29]. The P deficiency symptoms in P omitted treatments can be attributed to the minimal amounts of P available for plant uptake in all the soils which can be seen from the results of initial soil analysis. The poor root development in Ca omitted treatments can be associated with roles of calcium in root growth. Calcium is involved in cell growth, both at the plant terminal and the root tips. Absence of Ca leads to browning and dying of the root tips and thus leading to poorly developed root systems. Although all the growing tips are sensitive to Ca deficiency, those of the roots are affected more severely[34]. The significantly low concentration of other elements such as; P, B, Cu and Zn (Table 5) in Ca omitted treatments than the complete treatment can be attributed to the nutrients’ low uptake resulting from a poorly developed rooting system.

Nitrogen application to the nutrient solution was to help identify whether the poor performance in the various nutrient-omitted treatments was due to the nutrient in question or nitrogen deficiency. Addition of nitrogen to the nutrient solution led to the nitrogen concentrations in the plant tissues to fall within the required sufficiency ranges (3.5–5.5%)[29]. Although there were no to few nodules in most of the treatments, nitrogen added treatments did not have nodules in all the soils. This could be attributed to the ability of nitrogen to inhibit nodulation when supplied in large amounts [35]. Field experiments have shown a reduction in the nodule number when nitrogen was applied to soybean [35]and also in common bean, lima bean, green gram and lab lab [36]. It was also found out that addition of N (> 2.5 mM) inhibited nodulation in cowpea and soybean [37] under hydroponic solutions. There was low nodulation in most of the treatments despite the low N content. This could be attributed to the low pH of most of the soils which are not favourable for soybean nodulation and nitrogen fixation and thus nitrogen deficiencies could be noted.The low N concentration (below the sufficiency ranges– 3.5 to 5.5) in all the treatments except the plus N treatment (Table 5) can be attributed to the moderate initial N contents in soils and poor nodulation. This could also be attributed to the fact that maximum nitrogen fixation occurs at R3 to R5 stages of soybean growth [3]yet the plants in this study were harvested at V3 stage.

The insignificant difference between the lime applied treatment and the complete treatment in terms of shoot nutrient concentration may be due to the good supply of the nutrients from the nutrient solution in the lower pot that was not influenced by lime application.This may also be due to its ability to improve the pHof the soil which may have contributed to improved soil nutrient availability. Manganese concentrations in lime-added treatments were lower than all the other treatments. Lime reduces manganese concentration through mass action when applied to the soils by raising the soil pH. It was found out that liming reduced Mn concentrations in the soil, but the concentration in the leaves was sufficient but decreased with subsequent seasons under liming[38].

### Effects of the nutrient treatments on shoot dry weights

All the treatments were compared against the complete treatment in terms of shoot and root dry weights to determine their extent of limitation. This is because theoretically it is expected that the complete treatment should have the best performance because of the optimal nutrient conditions for growth. If the treatment had significantly lower SDWs than the complete treatment, it meant that the element was limiting[25].The poor performance of plants (in terms of shoot dry weights) growing in magnesium-omitted treatments may be attributed to the important roles played by magnesium in the plants. Magnesium is an important component of chlorophyll which helps in capturing energy from the sun for growth and development; Mg also plays an important role in activation of a number of enzymes important in protein synthesis and P reactions[39]. Magnesium deficiencies are widely reported and have resulted in ailments such as grass tetany in ruminant animals feeding on grasses with Mg deficiency[17]. Most field Mg deficiencies are induced by competing cations such as, K^+^, NH_4_^+^, Ca^2+^ and Mn^2+^ [40]. Magnesium deficiency symptoms have also been reported in the field trials. These are more pronounced in cases where K fertilizers were used when there was little Mg available in the soil[41].The poor performance of plants growing in minus K treatments in terms of the shoot dry weights indicated that K is limiting in these soils. It has been reported that relatively large amounts of K are required by high yielding soybean varieties[34]. This is because the dry matter yield, nodule parameters and the total nitrogen accumulation increases with increasing K supply. This can be associated with the improvement of nitrogenase activity and thus enhancing biological nitrogen fixation (BNF)[34,42].The low shoot dry weights could be attributed to the premature leaf fall.

The poor performance of plants growing in P omitted treatments in terms of shoot dry weights in all the soils except in Acrisols from Butere indicates that P is limiting in these soils. This agrees with many findings that P is one of the most limiting elements affecting soybean production in soils of western Kenya and this can be attributed to the widespread occurrence of soils with high P fixation capacity [2]. This can be the case in this study since the Acrisols and Ferralsols used were all acidic, suggesting the presence of iron and aluminum ions responsible for P fixation.Despite the poor root development in Ca-omitted treatments as explained earlier, the plants growing in this treatment were not significantly different from the complete treatment in terms of shoot dry weight in Ferralsols from Kakamega (Khwisero and Shikhulu Sub-locations). This can be attributed to the soil Ca status (moderate) especially for the Ferralsols from Kakamega (Khwisero and Shikhulu sub-locations) and Butula.

Potassium, Ca and Mg are the cations which are more prone to leaching from the soils. This occurs more in areas with heavy rainfall. Organic and sandy soils are prone to K leaching. Regular application of the fertilizers with these cations should be carried out to replenish the soils[34]. Extensive weathering of the Acrisols and Ferralsols has also led to the leaching of these cations over time[36].

Omission of S from the nutrient solution was not significantly different from the complete treatment in terms of shoot dry weights. Sulphur deficiency in plants is mainly indicated by chlorosis of young leaves. Sulphur is widely known for its functions in protein synthesis because it is a component of amino acids cysteine and methionine. Its availability therefore is mainly assessed by the analysis of the grains to establish their contents[40]. Micro-nutrients are very important in soybean nutrition. For instance, maximum production in leguminous plants can be obtained through effective nodulation and molybdenum application and this is well expressed in terms of yield and nitrogen concentration in the plant tissues[43]. This can be seen in the low (below the sufficiency ranges) accumulation of nitrogen in minus micro-nutrients treatments

Addition of nitrogen led to variable responses of soybean in the different soils. There was high foliage production in plants growing in nitrogen-added treatments in Ferralsols from Kakamega (Khwisero and Shikhulu sub-locations) and in Butula and thus led to significantly higher shoot dry weights compared to the complete treatment. From initial soil analysis, soils from Kakamega (Khwisero and Shikulu sub-locations) and Butula had higher nitrogen levels than those of Butere and Masaba Central although they were all at moderate levels (0.12 to 0.25). These low N levels might have led to the difference in plant performance. These can also be attributed to the moderate carbon levels in the soils from Butula and Kakamega. Nitrogen supply to plants increases leaf area and canopies. In dicots, impact of nitrogen supply in hydroponic systems on leaf growth is due to increased cell growth as observed by other earlier studies[39]. Results from field experiments have shown an increase in soybean’s and other grain legume’s dry matter and grain yields despite the reduction in nodulation upon the application of nitrogenous fertilizers [3,35,36]. This shows that nitrogen applied soybean plants can outcompete those grown on inoculation, unless management of the inoculated plants is improved.Those plants growing in Acrisols from Butere and Masaba central had less foliage and thus the shoot dry weights were not significantly different from those of the complete treatment. This can be attributed to the necrosis and scorching of the leaf tips and edges in these plants. This might be due to nickel deficiency thus leading to accumulation of urea in the leaves of N-added nutrient solution in these soils. High concentration of urea in plant leaves causes death of cells leading to necrotic lesions at leaf tips. Nickel which is a constituent of urease enzyme helps in hydrolysis of urea thus preventing its accumulation in plant leaves[44].

The significant increase of soybean shoot dry weights in response to lime application in Acrisols from Butere (Table 7) can be attributed to the ability of lime to significantly raise the soil pH in these soils (Table 9). It raises soil pH by replacing the H^+^ on the cation exchange complex with Ca^2+^. The H^+^ then combines with the hydroxyl ions (OH^-^) to form water. Calcium ions in the liming material also replaces the aluminum and manganese ions from the exchange sites thus increasing the cation saturation in the soil solution[45]. The rise in soil pH upon lime application might have contributed to the better performance of plants by increasing the availability of plant nutrients. Research in western Kenya has shown the positive influence of using lime in addressing the problem of soil acidity and therefore enhancing soil fertility [18]. Application of quick lime (CaO 21%) resulted in increase in soil pH and improved available P content in the soils of western Kenya[46]

### Effects of the nutrient treatments on Nutrient Sufficiency Quotients

The nutrient SQ is an index that can be used to assess the nutrient availability. It tells the ability of a soil to supply plant nutrients and thus can be used for fertilizer recommendations[23,47]. Sufficiency quotient which is the ratio of the relative growth rate between time points helps in indicating those nutrients which are present in insufficient amounts. The sufficiency quotients were therefore obtained by dividing the relative growth rates of the different treatments by the complete treatments. In the current study, all the treatments apart from the complete treatment should therefore have a sufficiency quotient of below 100%. This is because the complete treatment is provided with all the nutrients[25]. From the study, those nutrients with SQs less than 50% were considered to be more limiting. These included K and Magnesium in soils from Masaba, Shikhulu, Butere and Butula and P in soils from Butere, those more than 50% were considered to be less limiting and included Ca, micro-nutrients, P and S in Masaba, Ca, K and P in soils from Khwisero, micro-nutrients, P and S in soils from Shikhulu, Ca and S in soils from Butere and Ca, micro-nutrients and P in soils from Butula. Those above 100 were considered not limiting; micro-nutrients and S in Khwisero, Ca in soils from Shikhulu, micro-nutrients in soils from Butere and S in soils from Butula. The low SQs in K and Mg omitted treatments can be attributed to their premature leaf fall which led to their low shoot dry weights.

## Conclusions

The most limiting nutrients in Acrisols and Ferralsols studied for soybean production are K, Mg, and P. Magnesium and P were found to be the most limiting nutrients in both the Acrisols and Ferralsols; K was limiting in the Acrisols from Masaba central and Butere and Ferralsols from Kakamega (Shikhulu sub-location) and Butula. Lime application improved soil pH in all the soils and improved shoot dry weights over the complete treatment in all the Acrisols and Ferralsols from Kakamega (Shikhulu Sub-location) and in Butula. Addition of N to the nutrient solution significantly raised SDWs in soils from Kakamega (Khwisero and Shikhulu sub-location) and Butula and improved the shoot nitrogen concentration to the required sufficiency levels across the soils. This signifies the necessity of application of small quantities of N for initial soybean use. To increase soybean yields in Acrisols and Ferralsols of western Kenya, fertilizer formulations containing Mg, K, P, and Ca (complete fertilizer) and liming in combination with the inoculants in low pH soils are recommended. Further work should focus on establishing the response of soybean to combined application of limiting nutrients, formulating new fertilizer blends for legumes including establishing detailed economic viability of nutrient use for soybean production in these soils.
